# Weak convergence theorem for a class of split variational inequality problems and applications in a Hilbert space

**DOI:** 10.1186/s13660-017-1397-9

**Published:** 2017-05-25

**Authors:** Ming Tian, Bing-Nan Jiang

**Affiliations:** College of Science, Civil Aviation University of China, Tianjin, 300300 China

**Keywords:** 58E35, 47H09, 65J15, iterative method, extragradient method, weak convergence, variational inequality, monotone mapping, equilibrium problem, constrained convex minimization problem, split feasibility problem

## Abstract

In this paper, we consider the algorithm proposed in recent years by Censor, Gibali and Reich, which solves split variational inequality problem, and Korpelevich’s extragradient method, which solves variational inequality problems. As our main result, we propose an iterative method for finding an element to solve a class of split variational inequality problems under weaker conditions and get a weak convergence theorem. As applications, we obtain some new weak convergence theorems by using our weak convergence result to solve related problems in nonlinear analysis and optimization.

## Introduction

The variational inequality problem (VIP) is generated from the method of mathematical physics and nonlinear programming. It has considerable applications in many fields, such as physics, mechanics, engineering, economic decision, control theory and so on. Variational inequality is actually a system of partial differential equations. In 1964, Stampacchia [1] first introduced the VIP for modeling in the mechanics problem. The VIP was generated from mathematical physics equations early on because the Lax-Milgram theorem was extended from the Hilbert space to its nonempty closed convex subset, so we got the first existence and uniqueness theorem of VIP. In the 1990s, the VIP became more and more important in nonlinear analysis and optimization problem.

The VIP is to find an element $x^{*}\in C$ satisfying the inequality

1.1 $$ \bigl\langle Ax^{*}, x-x^{*} \bigr\rangle \geq0, \quad\forall x\in C, $$

where *C* is a nonempty closed convex subset of a real Hilbert space *H* and *A* is a mapping of *C* into *H*. The set of solutions of VIP (1.1) is denoted by $\operatorname {VI}(C,A)$.

We can easily show that $x^{*}\in \operatorname {VI}(C,A)$ is equivalent to

$$ x^{*}=P_{C}(I-\lambda A)x^{*}. $$

A simple iterative method algorithm for solving VIP (1.1) is the projection method

1.2 $$ x_{n+1}=P_{C}(I-\lambda A)x_{n} $$

for each $n\in\mathbb{N}$, where $P_{C}$ is the metric projection of *H* into *C* and *λ* is a positive real number. Indeed, if *A* is *η*-strongly monotone and *L*-Lipschitz continuous, $0<\lambda<\frac{2\eta}{L^{2}}$, then there exists a unique point in $\operatorname {VI}(C,A)$, and the sequence $\{x_{n}\}$ generated by (1.2) converges strongly to this point. If *A* is *α*-inverse strongly monotone, the solution of VIP (1.1) does not always exist. Assume that $\operatorname {VI}(C,A)$ is nonempty, $0<\lambda<2\alpha$, then $\operatorname {VI}(C,A)$ is a closed and convex subset of *H*. The sequence $\{x_{n}\}$ generated by (1.2) converges weakly to one of the points in $\operatorname {VI}(C,A)$. But if *A* is monotone and Lipschitz continuous, the sequence $\{x_{n}\}$ generated by (1.2) is not always convergent. So, we cannot use algorithm (1.2) to solve VIP (1.1).

In 1976, Korpelevich [2] introduced the following so-called extragradient method for solving VIP (1.1) when *A* is monotone and *k*-Lipschitz continuous in the finite-dimensional Euclidean space $\mathbb{R}^{n}$:

1.3 $$ \textstyle\begin{cases} y_{n}=P_{C}(x_{n}-\lambda Ax_{n}),\\ x_{n+1}=P_{C}(x_{n}-\lambda Ay_{n}), \end{cases} $$

for each $n\in\mathbb{N}$, where $\lambda\in(0,\frac{1}{k})$. The sequence $\{x_{n}\}$ converges to a point in $\operatorname {VI}(C,A)$.

The split feasibility problem (SFP) is also important in nonlinear analysis and optimization. In 1994, Censor and Elfving [3] first proposed it for modeling in medical image reconstruction. Recently, the SFP has been widely used in intensity-modulation therapy treatment planning.

The SFP is to find a point $x^{*}$ satisfying the conditions

1.4 $$ x^{*}\in C \quad \mbox{and} \quad Ax^{*}\in Q, $$

where *C* and *Q* are nonempty closed convex of real Hilbert spaces $H_{1}$ and $H_{2}$, respectively, and *A* is a bounded linear operator of $H_{1}$ into $H_{2}$.

Censor and Elfving used their algorithm to solve the SFP in the finite-dimensional Euclidean space $\mathbb{R}^{n}$. In 2001, Byrne [4] improved and extended Censor and Elfving’s algorithm and introduced a new method called *CQ* algorithm for solving the SFP (1.4)

1.5 $$ x_{n+1}=P_{C} \bigl(x_{n}-\gamma A^{*}(I-P_{Q})Ax_{n} \bigr) $$

for each $n\in\mathbb{N}$, where $0<\gamma<\frac{2}{ \Vert A \Vert ^{2}}$ and $A^{*}$ is the adjoint operator of *A*. The sequence $\{x_{n}\}$ generated by (1.5) converges weakly to a point which solves SFP (1.4).

Very recently, Censor et al. [5] combined the variational inequality problem and the split feasibility problem and proposed a new problem called split variational inequality problem (SVIP). The SVIP is to find a point $x^{*}$ satisfying

1.6 $$ x^{*}\in \operatorname {VI}(C,f) \quad \text{and} \quad Ax^{*}\in \operatorname {VI}(Q,g), $$

where *C* and *Q* are nonempty closed convex of the real Hilbert spaces $H_{1}$ and $H_{2}$, respectively, and *A* is a bounded linear operator of $H_{1}$ into $H_{2}$.

For solving SVIP (1.6), Censor, Gibali and Reich introduced the following algorithm:

1.7 $$ x_{n+1}=P_{C}(I-\lambda f) \bigl(x_{n}+\gamma A^{*} \bigl(P_{Q}(I-\lambda g)-I \bigr)Ax_{n} \bigr) $$

for each $n\in\mathbb{N}$. They obtained the following result.

### Theorem 1.1

[5, 6]

*Let* $A:H_{1}\rightarrow H_{2}$ *be a bounded linear operator*, $f:H_{1}\rightarrow H_{1}$ *and* $g:H_{2}\rightarrow H_{2}$ *be respectively* $\alpha_{1}$ *and* $\alpha _{2}$ *inverse strongly monotone operators and set* $\alpha:=\min\{\alpha_{1},\alpha _{2}\}$. *Assume that SVIP* (1.6) *is consistent*, $\gamma\in(0,\frac{1}{L})$ *with* *L* *being the spectral radius of the operator* $A^{*}A$, $\lambda\in (0,2\alpha)$ *and suppose that for all* $x^{*}$ *solving SVIP* (1.6),

1.8 $$ \bigl\langle f(x), P_{C}(I-\lambda f) (x)-x^{*} \bigr\rangle \geq0, \quad\forall x\in H_{1}. $$

*Then the sequence* $\{x_{n}\}$ *generated by* (1.7) *converges weakly to a solution of SVIP* (1.6).

In this paper, based on the work by Censor et al. combined with Korpelevich’s extragradient method and Byrne’s *CQ* algorithm, we propose an iterative method for finding an element to solve a class of split variational inequality problems under weaker conditions and get a weak convergence theorem. As applications, we obtain some new weak convergence theorems by using our weak convergence result to solve related problems in nonlinear analysis and optimization.

## Preliminaries

In this section, we introduce some mathematical symbols, definitions, lemmas and corollaries which can be used in the proofs of our main results.

Let $\mathbb{N}$ and $\mathbb{R}$ be the sets of positive integers and real numbers, respectively. Let *H* be a real Hilbert space with the inner product $\langle\cdot,\cdot\rangle $ and the norm $\Vert \cdot \Vert $. Let $\{x_{n}\}$ be a sequence in *H*, we denote the sequence $\{x_{n}\}$ converging weakly to *x* by ‘$x_{n}\rightharpoonup x$’ and the sequence $\{x_{n}\}$ converging strongly to *x* by ‘$x_{n}\rightarrow x$’. *z* is called a weak cluster point of $\{x_{n}\}$ if there exists a subsequence $\{x_{n_{i}}\}$ of $\{x_{n}\}$ converging weakly to *z*. The set of all weak cluster points of $\{x_{n}\}$ is denoted by $\omega_{w}(x_{n})$. A fixed point of the mapping $T:H\rightarrow H$ is a point $x\in H$ such that $Tx=x$. The set of all fixed points of the mapping *T* is denoted by $\operatorname {Fix}(T)$.

We introduce some useful operators.

### Definition 2.1

[7]

Let *T*, $A:H\rightarrow H$ be nonlinear operators.

(i) *T* is nonexpansive if
  $$ \Vert Tx-Ty \Vert \leq \Vert x-y \Vert , \quad \forall x,y\in H. $$
(ii) *T* is firmly nonexpansive if
  $$ \langle Tx-Ty, x-y\rangle\geq \Vert Tx-Ty \Vert ^{2}, \quad \forall x,y\in H. $$
(iii) *T* is *L*-Lipschitz continuous, with $L>0$, if
  $$ \Vert Tx-Ty \Vert \leq L \Vert x-y \Vert , \quad \forall x,y\in H. $$
(iv) *T* is *α*-averaged if
  $$ T=(1-\alpha)I+\alpha S, $$
  where $\alpha\in(0,1)$ and $S:H\rightarrow H$ is nonexpansive. In particular, a firmly nonexpansive mapping is $\frac {1}{2}$-averaged.
(v) *A* is monotone if
  $$ \langle Ax-Ay, x-y\rangle\geq0, \quad\forall x,y\in H. $$
(vi) *A* is *η*-strongly monotone, with $\eta>0$, if
  $$ \langle Ax-Ay, x-y\rangle\geq\eta \Vert x-y \Vert ^{2}, \quad \forall x,y\in H. $$
(vii) *A* is *v*-inverse strongly monotone (*v*-ism), with $v>0$, if
  $$ \langle Ax-Ay, x-y\rangle\geq v \Vert Ax-Ay \Vert ^{2}, \quad \forall x,y\in H. $$

We can easily show that if *T* is a nonexpansive mapping and assume that $\operatorname {Fix}(T)$ is nonempty, then $\operatorname {Fix}(T)$ is a closed convex subset of *H*.

We need the propositions of averaged mappings and inverse strongly monotone mappings.

### Lemma 2.2

[7]

*We have the following propositions*.

(i) *T* *is nonexpansive if and only if the complement* $I-T$ *is* $\frac {1}{2}$-*ism*.
(ii) *If* *T* *is* *v*-*ism and* $\gamma>0$, *then* *γT* *is* $\frac {v}{\gamma}$-*ism*.
(iii) *T* *is averaged if and only if the complement* $I-T$ *is* *v*-*ism for some* $v>\frac{1}{2}$. *Indeed*, *for* $\alpha\in(0,1)$, *T* *is* *α*-*averaged if and only if* $I-T$ *is* $\frac{1}{2\alpha}$-*ism*.
(iv) *If* $T_{1}$ *is* $\alpha_{1}$-*averaged and* $T_{2}$ *is* $\alpha_{2}$-*averaged*, *where* $\alpha_{1}$, $\alpha_{2}\in(0,1)$, *then the composite* $T_{1}T_{2}$ *is* *α*-*averaged*, *where* $\alpha=\alpha_{1}+\alpha_{2}-\alpha_{1}\alpha _{2}$.
(v) *If* $T_{1}$ *and* $T_{2}$ *are averaged and have a common fixed point*, *then* $\operatorname {Fix}(T_{1}T_{2})=\operatorname {Fix}(T_{1})\cap \operatorname {Fix}(T_{2})$.

### Lemma 2.3

[8]

*Let* $H_{1}$ *and* $H_{2}$ *be real Hilbert spaces*. *Let* $A:H_{1}\rightarrow H_{2}$ *be a bounded linear operator with* $A\neq0$ *and* $T:H_{2}\rightarrow H_{2}$ *be a nonexpansive mapping*. *Then* $A^{*}(I-T)A$ *is* $\frac{1}{2 \Vert A \Vert ^{2}}$-*ism*.

Let *C* be a nonempty closed convex subset of *H*. For each $x\in H$, there exists a unique nearest point in *C*, denoted by $P_{C}x$, such that

$$ \Vert x-P_{C}x \Vert \leq \Vert x-y \Vert , \quad \forall y \in C. $$

$P_{C}$ is called the metric projection of *H* into *C*. We know that $P_{C}$ is firmly nonexpansive.

### Lemma 2.4

[9]

*Let* *C* *be a nonempty closed convex subset of a real Hilbert space* *H*. *Given* $x\in H$ *and* $z\in C$. *Then* $z=P_{C}x$ *if and only if there holds the inequality*

$$ \langle x-z, z-y\rangle\geq0, \quad\forall y\in C. $$

### Lemma 2.5

[9]

*Let* *C* *be a nonempty closed convex subset of a real Hilbert space* *H*. *Given* $x\in H$ *and* $z\in C$. *Then* $z=P_{C}x$ *if and only if there holds the inequality*

$$ \Vert x-y \Vert ^{2}\geq \Vert x-z \Vert ^{2}+ \Vert y-z \Vert ^{2}, \quad\forall y\in C. $$

We introduce some definitions and propositions about set-valued mappings.

### Definition 2.6

[8]

Let *B* be a set-valued mapping of *H* into $2^{H}$. We denote the effective domain of *B* by $D(B)$, $D(B)$ is defined by

$$ D(B)=\{x\in H: Bx\neq\emptyset\}. $$

A set-valued mapping *B* is called monotone if

$$ \langle x-y, u-v\rangle\geq0, \quad\forall x,y\in D(B), u\in Bx, v\in By. $$

A monotone mapping *B* is called maximal if its graph is not properly contained in the graph of any other monotone mappings on $D(B)$.

In fact, the definition of the maximal monotone mapping is not very convenient to use. We usually use a property of the maximal monotone mapping: A monotone mapping *B* is maximal if and only if for $(x,u)\in H\times H$, $\langle x-y, u-v\rangle\geq0$ for each $(y,v)\in G(B)$ implies $u\in Bx$

For a maximal monotone mapping *B*, we define its resolvent $J_{r}$ by

$$ J_{r}:=(I+rB)^{-1}:H\rightarrow D(B), $$

where $r>0$. We know that $J_{r}$ is firmly nonexpansive and $\operatorname {Fix}(J_{r})=B^{-1}0$ for each $r>0$.

For a nonempty closed convex subset *C*, the normal cone to *C* at $x\in C$ is defined by

$$ N_{C}x= \bigl\{ z\in H: \langle z, y-x\rangle\leq0, \forall y\in C \bigr\} . $$

It can be easily shown that $N_{C}$ is a maximal monotone mapping and its resolvent is $P_{C}$.

### Lemma 2.7

[10]

*Let* *C* *be a nonempty closed convex subset of a real Hilbert space* *H*. *Let* *A* *be a monotone and* *k*-*Lipschitz continuous mapping of* *C* *into* *H*. *Define*

$$ Bv= \textstyle\begin{cases} Av+N_{C}v, &\forall v\in C,\\ \emptyset,&\forall v\notin C. \end{cases} $$

*Then* *B* *is maximal monotone and* $0\in Bv$ *if and only if* $v\in \operatorname {VI}(C,A)$.

### Lemma 2.8

[10]

*Let* $H_{1}$ *and* $H_{2}$ *be real Hilbert spaces*. *Let* $B:H_{1}\rightarrow 2^{H_{1}}$ *be a maximal monotone mapping*, *and let* $J_{r}$ *be the resolvent of* *B* *for* $r>0$. *Let* $T:H_{2}\rightarrow H_{2}$ *be a nonexpansive mapping*, *and let* $A:H_{1}\rightarrow H_{2}$ *be a bounded linear operator*. *Suppose that* $B^{-1}0\cap A^{-1}\operatorname {Fix}(T)\neq\emptyset$. *Let* $r,\gamma>0$ *and* $z\in H_{1}$. *Then the following are equivalent*:

(i) $z=J_{r}(I-\gamma A^{*}(I-T)A)z$;
(ii) $0\in A^{*}(I-T)Az+Bz$;
(iii) $z\in B^{-1}0\cap A^{-1}\operatorname {Fix}(T)$.

### Corollary 2.9

*Let* $H_{1}$ *and* $H_{2}$ *be real Hilbert spaces*. *Let* *C* *be a nonempty closed convex subset of* $H_{1}$. *Let* $T:H_{2}\rightarrow H_{2}$ *be a nonexpansive mapping*, *and let* $A:H_{1}\rightarrow H_{2}$ *be a bounded linear operator*. *Suppose that* $C\cap A^{-1}\operatorname {Fix}(T)\neq \emptyset$. *Let* $\gamma>0$ *and* $z\in H_{1}$. *Then the following are equivalent*:

(i) $z=P_{C}(I-\gamma A^{*}(I-T)A)z$;
(ii) $0\in A^{*}(I-T)Az+N_{C}z$;
(iii) $z\in C\cap A^{-1}\operatorname {Fix}(T)$.

### Proof

Putting $B=N_{C}$ in Lemma 2.8, we obtain the desired result. □

We also need the following lemmas.

### Lemma 2.10

[11]

*Let* *H* *be a real Hilbert space and* $T:H\rightarrow H$ *be a nonexpansive mapping with* $\operatorname {Fix}(T)\neq\emptyset$. *If* $\{x_{n}\}$ *is a sequence in* *H* *converging weakly to* *x* *and if* $\{(I-T)x_{n}\}$ *converges strongly to* *y*, *then* $(I-T)x=y$.

### Lemma 2.11

[12]

*Let* *C* *be a nonempty closed convex subset of a real Hilbert space* *H*. *Let* $\{x_{n}\}$ *be a sequence in* *H* *satisfying the properties*:

(i) $\lim_{n\rightarrow\infty} \Vert x_{n}-u \Vert $ *exists for each* $u\in C$;
(ii) $\omega_{w}(x_{n})\subset C$.

*Then* $\{x_{n}\}$ *converges weakly to a point in C*.

### Lemma 2.12

[13]

*Let* *C* *be a nonempty closed convex subset of a real Hilbert space* *H*. *Let* $\{x_{n}\}$ *be a sequence in* *H*. *Suppose that*

$$ \Vert x_{n+1}-u \Vert \leq \Vert x_{n}-u \Vert , \quad\forall u\in C, $$

*for every* $n=0,1,2,\ldots$ . *Then the sequence* $\{P_{C}x_{n}\}$ *converges strongly to a point in C*.

## Main results

In this section, based on Censor, Gibali and Reich’s recent work and combining it with Byrne’s *CQ* algorithm and Korpelevich’s extragradient method, we propose a new iterative method for solving a class of the SVIP under weaker conditions in a Hilbert space.

First, we consider a class of the generalized split feasibility problems (GSFP): finding an element that solves a variational inequality problem such that its image under a given bounded linear operator is in a fixed point set of a nonexpansive mapping, i.e., find $x^{*}$ satisfying

3.1 $$ x^{*}\in \operatorname {VI}(C,f) \quad \text{and} \quad Ax^{*}\in \operatorname {Fix}(T). $$

### Theorem 3.1

*Let* $H_{1}$ *and* $H_{2}$ *be real Hilbert spaces*. *Let* *C* *be a nonempty closed convex subset of* $H_{1}$. *Let* $A:H_{1}\rightarrow H_{2}$ *be a bounded linear operator such that* $A\neq0$, $f:C\rightarrow H_{1}$ *be a monotone and* *k*-*Lipschitz continuous mapping and* $T:H_{2}\rightarrow H_{2}$ *be a nonexpansive mapping*. *Setting* $\Gamma=\{z\in \operatorname {VI}(C,f): Az\in \operatorname {Fix}(T)\}$, *assume that* $\Gamma\neq\emptyset$. *Let the sequences* $\{x_{n}\}$, $\{y_{n}\}$ *and* $\{t_{n}\}$ *be generated by* $x_{1}=x\in C$ *and*

3.2 $$ \textstyle\begin{cases} y_{n}=P_{C}(x_{n}-\gamma_{n}A^{*}(I-T)Ax_{n}),\\ t_{n}=P_{C}(y_{n}-\lambda_{n}f(y_{n})),\\ x_{n+1}=P_{C}(y_{n}-\lambda_{n}f(t_{n})), \end{cases} $$

*for each* $n\in\mathbb{N}$, *where* $\{\gamma_{n}\}\subset[a,b]$ *for some* $a,b\in(0,\frac{1}{ \Vert A \Vert ^{2}})$ *and* $\{\lambda_{n}\} \subset[c,d]$ *for some* $c,d\in(0,\frac{1}{k})$. *Then the sequence* $\{x_{n}\}$ *converges weakly to a point* $z\in\Gamma$, *where* $z=\lim_{n\rightarrow \infty} P_{\Gamma}x_{n}$.

### Proof

From Lemma 2.2(ii), (iii), (iv) and Lemma 2.3, we can easily know that $P_{C}(I-\gamma_{n}A^{*}(I-T)A)$ is $\frac{1+\gamma_{n} \Vert A \Vert ^{2}}{2}$-averaged. So, $y_{n}$ can be rewritten as

3.3 $$ y_{n}=(1-\alpha_{n})x_{n}+ \alpha_{n}V_{n}x_{n}, $$

where $\alpha_{n}=\frac{1+\gamma_{n} \Vert A \Vert ^{2}}{2}$ and $V_{n}$ is a nonexpansive mapping for each $n\in\mathbb{N}$.

Let $u\in\Gamma$, we have

3.4 $$\begin{aligned}& \Vert y_{n}-u \Vert ^{2} \\& \quad = \bigl\Vert (1-\alpha_{n}) (x_{n}-u)+ \alpha_{n}(V_{n}x_{n}-u) \bigr\Vert ^{2} \\& \quad = (1-\alpha_{n}) \Vert x_{n}-u \Vert ^{2}+ \alpha_{n} \Vert V_{n}x_{n}-u \Vert ^{2} -\alpha_{n}(1-\alpha_{n}) \Vert x_{n}-V_{n}x_{n} \Vert ^{2} \\& \quad \leq \Vert x_{n}-u \Vert ^{2}- \alpha_{n}(1- \alpha_{n}) \Vert x_{n}-V_{n}x_{n} \Vert ^{2} \\& \quad \leq \Vert x_{n}-u \Vert ^{2}. \end{aligned}$$

So, we obtain that

3.5 $$ \alpha_{n}(1-\alpha_{n}) \Vert x_{n}-V_{n}x_{n} \Vert ^{2}\leq \Vert x_{n}-u \Vert ^{2}- \Vert y_{n}-u \Vert ^{2}. $$

On the other hand, from Lemma 2.5, we have

3.6 $$\begin{aligned}& \Vert x_{n+1}-u \Vert ^{2} \\& \quad \leq \bigl\Vert y_{n}-\lambda_{n}f(t_{n})-u \bigr\Vert ^{2}- \bigl\Vert y_{n}-\lambda_{n}f(t_{n})-x_{n+1} \bigr\Vert ^{2} \\& \quad = \Vert y_{n}-u \Vert ^{2}- \Vert y_{n}-x_{n+1} \Vert ^{2}+2\lambda_{n} \bigl\langle f(t_{n}), u-x_{n+1} \bigr\rangle \\& \quad = \Vert y_{n}-u \Vert ^{2}- \Vert y_{n}-x_{n+1} \Vert ^{2}+2\lambda_{n} \bigl( \bigl\langle f(t_{n}), u-t_{n} \bigr\rangle \\& \quad \quad{} + \bigl\langle f(t_{n}), t_{n}-x_{n+1} \bigr\rangle \bigr) \\& \quad = \Vert y_{n}-u \Vert ^{2}- \Vert y_{n}-x_{n+1} \Vert ^{2}+2\lambda_{n} \bigl( \bigl\langle f(t_{n})-f(u), u-t_{n} \bigr\rangle \\& \quad \quad{} + \bigl\langle f(u), u-t_{n} \bigr\rangle + \bigl\langle f(t_{n}), t_{n}-x_{n+1} \bigr\rangle \bigr) \\& \quad \leq \Vert y_{n}-u \Vert ^{2}- \Vert y_{n}-x_{n+1} \Vert ^{2}+2\lambda_{n} \bigl\langle f(t_{n}), t_{n}-x_{n+1} \bigr\rangle \\& \quad = \Vert y_{n}-u \Vert ^{2}- \Vert y_{n}-t_{n} \Vert ^{2}-2\langle y_{n}-t_{n}, t_{n}-x_{n+1}\rangle \\& \quad\quad{} - \Vert t_{n}-x_{n+1} \Vert ^{2}+2 \lambda_{n} \bigl\langle f(t_{n}), t_{n}-x_{n+1} \bigr\rangle \\& \quad = \Vert y_{n}-u \Vert ^{2}- \Vert y_{n}-t_{n} \Vert ^{2}- \Vert t_{n}-x_{n+1} \Vert ^{2} \\& \quad\quad{} +2 \bigl\langle y_{n}-\lambda_{n}f(t_{n})-t_{n}, x_{n+1}-t_{n} \bigr\rangle . \end{aligned}$$

Then, from Lemma 2.4, we obtain that

3.7 $$\begin{aligned}& \bigl\langle y_{n}-\lambda_{n}f(t_{n})-t_{n}, x_{n+1}-t_{n} \bigr\rangle \\& \quad = \bigl\langle y_{n}-\lambda_{n}f(y_{n})-t_{n}, x_{n+1}-t_{n} \bigr\rangle + \bigl\langle \lambda_{n}f(y_{n})- \lambda_{n}f(t_{n}), x_{n+1}-t_{n} \bigr\rangle \\& \quad \leq \bigl\langle \lambda_{n}f(y_{n})- \lambda_{n}f(t_{n}), x_{n+1}-t_{n} \bigr\rangle \\& \quad = \lambda_{n} \bigl\langle f(y_{n})-f(t_{n}), x_{n+1}-t_{n} \bigr\rangle \\& \quad \leq \lambda_{n} \bigl\Vert f(y_{n})-f(t_{n}) \bigr\Vert \Vert x_{n+1}-t_{n} \Vert \\& \quad \leq \lambda_{n}k \Vert y_{n}-t_{n} \Vert \Vert x_{n+1}-t_{n} \Vert . \end{aligned}$$

So, we have

3.8 $$\begin{aligned}& \Vert x_{n+1}-u \Vert ^{2} \\& \quad \leq \Vert y_{n}-u \Vert ^{2}- \Vert y_{n}-t_{n} \Vert ^{2}- \Vert t_{n}-x_{n+1} \Vert ^{2} \\& \quad\quad{} +2\lambda_{n}k \Vert y_{n}-t_{n} \Vert \Vert x_{n+1}-t_{n} \Vert \\& \quad \leq \Vert y_{n}-u \Vert ^{2}- \Vert y_{n}-t_{n} \Vert ^{2}- \Vert t_{n}-x_{n+1} \Vert ^{2} \\& \quad \quad{} +\lambda_{n}^{2}k^{2} \Vert y_{n}-t_{n} \Vert ^{2}+ \Vert x_{n+1}-t_{n} \Vert ^{2} \\& \quad \leq \Vert y_{n}-u \Vert ^{2}+ \bigl( \lambda_{n}^{2}k^{2}-1 \bigr) \Vert y_{n}-t_{n} \Vert ^{2} \\& \quad \leq \Vert y_{n}-u \Vert ^{2} \\& \quad \leq \Vert x_{n}-u \Vert ^{2}. \end{aligned}$$

Therefore, there exists

3.9 $$ c=\lim_{n\rightarrow\infty} \Vert x_{n}-u \Vert , $$

and the sequence $\{x_{n}\}$ is bounded. From (3.5), we also get

3.10 $$ \alpha_{n}(1-\alpha_{n}) \Vert x_{n}-V_{n}x_{n} \Vert ^{2}\leq \Vert x_{n}-u \Vert ^{2}- \Vert x_{n+1}-u \Vert ^{2}. $$

Hence

3.11 $$ x_{n}-V_{n}x_{n}\rightarrow0, \quad n\rightarrow\infty. $$

From (3.3), we can get

3.12 $$ x_{n}-y_{n}\rightarrow0, \quad n\rightarrow \infty. $$

From (3.8), we also get

3.13 $$ \Vert y_{n}-t_{n} \Vert ^{2} \leq\frac{1}{1-\lambda_{n}^{2}k^{2}} \bigl( \Vert x_{n}-u \Vert ^{2}- \Vert x_{n+1}-u \Vert ^{2} \bigr). $$

So,

3.14 $$ y_{n}-t_{n}\rightarrow0, \quad n\rightarrow \infty. $$

From (3.12) and (3.14), we have

3.15 $$ x_{n}-t_{n}\rightarrow0, \quad n\rightarrow \infty. $$

Notice that $P_{C}$ is firmly nonexpansive, so

3.16 $$\begin{aligned}& \Vert x_{n+1}-t_{n} \Vert \\& \quad = \bigl\Vert P_{C} \bigl(y_{n}- \lambda_{n}f(t_{n}) \bigr)-P_{C} \bigl(y_{n}-\lambda _{n}f(y_{n}) \bigr) \bigr\Vert \\& \quad \leq \bigl\Vert y_{n}-\lambda_{n}f(t_{n})-y_{n}+ \lambda _{n}f(y_{n}) \bigr\Vert \\& \quad = \bigl\Vert \lambda_{n}f(t_{n})- \lambda_{n}f(y_{n}) \bigr\Vert \\& \quad = \lambda_{n} \bigl\Vert f(t_{n})-f(y_{n}) \bigr\Vert \\& \quad \leq \lambda_{n}k \Vert t_{n}-y_{n} \Vert . \end{aligned}$$

We obtain

3.17 $$ x_{n+1}-t_{n}\rightarrow0, \quad n\rightarrow \infty. $$

From (3.15) and (3.17), we have

3.18 $$ x_{n+1}-x_{n}\rightarrow0, \quad n\rightarrow \infty. $$

Since $\{x_{n}\}$ is bounded, for each $z\in\omega_{w}(x_{n})$, there exists a subsequence $\{x_{n_{i}}\}$ of $\{x_{n}\}$ converging weakly to *z*. Without loss of generality, the subsequence $\{\gamma_{n_{i}}\}$ of $\{\gamma_{n}\}$ converges to a point $\hat{\gamma}\in(0,\frac{1}{ \Vert A \Vert ^{2}})$. Since $A^{*}(I-T)A$ is inverse strongly monotone, we know that $\{A^{*}(I-T)Ax_{n_{i}}\}$ is bounded. Since $P_{C}$ is firmly nonexpansive,

$$ \bigl\Vert P_{C} \bigl(I-\gamma_{n_{i}}A^{*}(I-T)A \bigr)x_{n_{i}}-P_{C} \bigl(I-\hat {\gamma}A^{*}(I-T)A \bigr)x_{n_{i}} \bigr\Vert \leq \vert \gamma_{n_{i}}-\hat{ \gamma} \vert \bigl\Vert A^{*}(I-T)Ax_{n_{i}} \bigr\Vert . $$

From $\gamma_{n_{i}}\rightarrow\hat{\gamma}$, we have

3.19 $$ P_{C} \bigl(I-\gamma_{n_{i}}A^{*}(I-T)A \bigr)x_{n_{i}}-P_{C} \bigl(I-\hat{\gamma }A^{*}(I-T)A \bigr)x_{n_{i}} \rightarrow0, \quad i\rightarrow\infty. $$

From (3.12), we have

3.20 $$ x_{n_{i}}-P_{C} \bigl(I-\gamma_{n_{i}}A^{*}(I-T)A \bigr)x_{n_{i}}\rightarrow0, \quad i\rightarrow\infty. $$

Since

$$\begin{aligned}& \bigl\Vert x_{n_{i}}-P_{C} \bigl(I-\hat{ \gamma}A^{*}(I-T)A \bigr)x_{n_{i}} \bigr\Vert \\& \quad \leq \bigl\Vert x_{n_{i}}-P_{C} \bigl(I-\gamma _{n_{i}}A^{*}(I-T)A \bigr)x_{n_{i}} \bigr\Vert \\& \quad\quad{} + \bigl\Vert P_{C} \bigl(I-\gamma _{n_{i}}A^{*}(I-T)A \bigr)x_{n_{i}}-P_{C} \bigl(I-\hat{\gamma }A^{*}(I-T)A \bigr)x_{n_{i}} \bigr\Vert , \end{aligned}$$

we have

3.21 $$ x_{n_{i}}-P_{C} \bigl(I-\hat{ \gamma}A^{*}(I-T)A \bigr)x_{n_{i}}\rightarrow0, \quad i \rightarrow \infty. $$

From Lemma 2.10, we obtain that

$$ z\in \operatorname {Fix}\bigl(P_{C} \bigl(I-\hat{\gamma}A^{*}(I-T)A \bigr) \bigr). $$

From Corollary 2.9, we obtain

3.22 $$ z\in C\cap A^{-1}\operatorname {Fix}(T). $$

Now, we show that $z\in \operatorname {VI}(C,f)$. From (3.12), (3.15) and (3.18), we have $y_{n_{i}}\rightharpoonup z$, $t_{n_{i}}\rightharpoonup z$ and $x_{n_{i}+1}\rightharpoonup z$.

Let

$$ Bv= \textstyle\begin{cases} f(v)+N_{C}v, &\forall v\in C,\\ \emptyset,&\forall v\notin C. \end{cases} $$

From Lemma 2.7, we know that *B* is maximal monotone and $0\in Bv$ if and only if $v\in \operatorname {VI}(C,f)$.

For each $(v,w)\in G(B)$, we have

$$ w\in Bv=f(v)+N_{C}v. $$

Hence

$$ w-f(v)\in N_{C}v. $$

So, we obtain that

3.23 $$ \bigl\langle v-p, w-f(v) \bigr\rangle \geq0, \quad\forall p\in C. $$

On the other hand, from $v\in C$ and

$$ x_{n+1}=P_{C} \bigl(y_{n}-\lambda_{n}f(t_{n}) \bigr), $$

we get

$$ \bigl\langle y_{n}-\lambda_{n}f(t_{n})-x_{n+1}, x_{n+1}-v \bigr\rangle \geq0 $$

and hence

3.24 $$ \biggl\langle v-x_{n+1}, \frac{x_{n+1}-y_{n}}{\lambda_{n}}+f(t_{n}) \biggr\rangle \geq0. $$

Therefore, from (3.23) and (3.24), we obtain that

3.25 $$\begin{aligned}& \langle v-x_{n_{i}+1}, w\rangle \\& \quad \geq \bigl\langle v-x_{n_{i}+1}, f(v) \bigr\rangle \\& \quad \geq \bigl\langle v-x_{n_{i}+1}, f(v) \bigr\rangle - \biggl\langle v-x_{n_{i}+1}, \frac{x_{n_{i}+1}-y_{n_{i}}}{\lambda _{n_{i}}}+f(t_{n_{i}}) \biggr\rangle \\& \quad = \bigl\langle v-x_{n_{i}+1}, f(v)-f(t_{n_{i}}) \bigr\rangle - \biggl\langle v-x_{n_{i}+1}, \frac{x_{n_{i}+1}-y_{n_{i}}}{\lambda _{n_{i}}} \biggr\rangle \\& \quad = \bigl\langle v-x_{n_{i}+1}, f(v)-f(x_{n_{i}+1}) \bigr\rangle + \bigl\langle v-x_{n_{i}+1}, f(x_{n_{i}+1})-f(t_{n_{i}}) \bigr\rangle \\& \quad\quad{} - \biggl\langle v-x_{n_{i}+1}, \frac{x_{n_{i}+1}-y_{n_{i}}}{\lambda _{n_{i}}} \biggr\rangle \\& \quad \geq \bigl\langle v-x_{n_{i}+1}, f(x_{n_{i}+1})-f(t_{n_{i}}) \bigr\rangle - \biggl\langle v-x_{n_{i}+1}, \frac{x_{n_{i}+1}-y_{n_{i}}}{\lambda_{n_{i}}} \biggr\rangle . \end{aligned}$$

As $i\rightarrow\infty$, we have

3.26 $$ \langle v-z, w\rangle\geq0. $$

Since *B* is maximal monotone, we have $0\in Bz$ and hence $z\in \operatorname {VI}(C,f)$. So, we obtain that

3.27 $$ \omega_{w}(x_{n})\subset\Gamma. $$

From Lemma 2.11, we get

3.28 $$ x_{n}\rightharpoonup z\in\Gamma, $$

and from Lemma 2.12, we obtain

3.29 $$ z=\lim_{n\rightarrow\infty}P_{\Gamma}x_{n}. $$

 □

### Remark 3.2

(i) If $f=0$ and $T=P_{Q}$, problem (3.1) reduces to the SFP introduced by Censor and Elfving, and the algorithm in Theorem 3.1 reduces to Byrne’s *CQ* algorithm for solving the SFP.
(ii) If $T=I$, problem (3.1) reduces to the VIP, and the algorithm in Theorem 3.1 reduces to Korpelevich’s extragradient method for solving the VIP with a monotone mapping.
(iii) If $A=I$, $f=0$ and $C=H_{1}$, problem (3.1) reduces to the fixed point problem of a nonexpansive mapping.

Now, we consider SVIP (1.6).

### Theorem 3.3

*Let* $H_{1}$ *and* $H_{2}$ *be real Hilbert spaces*. *Let* *C* *be a nonempty closed convex subset of* $H_{1}$ *and* *Q* *be a nonempty closed convex subset of* $H_{2}$. *Let* $A:H_{1}\rightarrow H_{2}$ *be a bounded linear operator such that* $A\neq0$, *let* $f:C\rightarrow H_{1}$ *be a monotone and* *k*-*Lipschitz continuous mapping and* $g:H_{2}\rightarrow H_{2}$ *be an* *α*-*inverse strongly monotone mapping*. *Setting* $\Gamma=\{z\in \operatorname {VI}(C,f): Az\in \operatorname {VI}(Q,g)\}$, *assume that* $\Gamma\neq\emptyset$. *Let the sequences* $\{x_{n}\}$, $\{y_{n}\}$ *and* $\{t_{n}\}$ *be generated by* $x_{1}=x\in C$ *and*

3.30 $$ \textstyle\begin{cases} y_{n}=P_{C}(x_{n}-\gamma_{n}A^{*}(I-P_{Q}(I-\mu g))Ax_{n}),\\ t_{n}=P_{C}(y_{n}-\lambda_{n}f(y_{n})),\\ x_{n+1}=P_{C}(y_{n}-\lambda_{n}f(t_{n})), \end{cases} $$

*for each* $n\in\mathbb{N}$, *where* $\{\gamma_{n}\}\subset[a,b]$ *for some* $a,b\in(0,\frac{1}{ \Vert A \Vert ^{2}})$, $\{\lambda_{n}\} \subset[c,d]$ *for some* $c,d\in(0,\frac{1}{k})$, $\mu\in(0,2\alpha)$. *Then the sequence* $\{x_{n}\}$ *converges weakly to a point* $z\in\Gamma$, *where* $z=\lim_{n\rightarrow \infty} P_{\Gamma}x_{n}$.

### Proof

From Lemma 2.4, we can easily know that $z\in \operatorname {VI}(Q,g)$ if and only if $z=P_{Q}(I-\mu g)z$ for $\mu>0$, and for $\mu\in(0,2\alpha)$, $P_{Q}(I-\mu g)$ is nonexpansive. Putting $T=P_{Q}(I-\mu g)$ in Theorem 3.1, we get the desired result. □

### Remark 3.4

(i) If $f=0$ and $g=0$, SVIP (1.6) reduces to the SFP introduced by Censor and Elfving, and the algorithm in Theorem 3.3 reduces to Byrne’s CQ algorithm for solving the SFP.
(ii) If $f=0$, $C=H_{1}$ and $A=I$, SVIP (1.6) reduces to the VIP and the algorithm in Theorem 3.3 reduces to (1.2) for solving the VIP with an inverse strongly monotone mapping.
(iii) If $g=0$ and $Q=H_{2}$, SVIP (1.6) reduces to the VIP and the algorithm in Theorem 3.1 reduces to Korpelevich’s extragradient method for solving the VIP with a monotone mapping.

## Application

In this part, some applications which are useful in nonlinear analysis and optimization problems in a Hilbert space will be introduced. This section deals with some weak convergence theorems for some generalized split feasibility problems (GSFP) and some related problems by Theorem 3.1 and Theorem 3.3.

Let *C* be a nonempty closed convex subset of a real Hilbert space *H*, and let $F:C\times C\rightarrow\mathbb{R}$ be a bifunction. Consider the equilibrium problem, i.e., find $x^{*}$ satisfying

4.1 $$ F \bigl(x^{*},y \bigr)\geq0, \quad\forall y\in C. $$

We denote the set of solutions of equilibrium problem (4.1) by $\operatorname {EP}(F)$. For solving equilibrium problem (4.1), we assume that *F* satisfies the following properties:

1. $F(x,x)$=0 for all $x\in C$;
2. *F* is monotone, i.e., $F(x,y)+F(y,x)\leq0$ for all $x,y\in C$;
3. for each $x, y, z\in C, \lim_{t\downarrow0}F(tz+(1-t)x,y)\leq F(x,y)$;
4. for each $x\in C, y\mapsto F(x,y)$ is convex and lower semicontinuous.

Then we have the following lemmas.

### Lemma 4.1

[14]

*Let* *C* *be a nonempty closed convex subset of a real Hilbert space* *H*, *and let* *F* *be a bifunction of* $C\times C$ *into* $\mathbb{R}$ *satisfying the properties* (A1)-(A4). *Let* *r* *be a positive real number and* $x\in H$. *Then there exists* $z\in C$ *such that*

$$ F(z,y)+\frac{1}{r}\langle y-z, z-x\rangle\geq0, \quad\forall y\in C. $$

### Lemma 4.2

[15]

*Assume that* $F:C\times C\rightarrow\mathbb{R}$ *is a bifunction satisfying the properties* (A1)-(A4). *For* $r>0$ *and* $x\in H$, *define the resolvent* $T_{r}:H\rightarrow C$ *of* *F* *by*

$$ T_{r}x= \biggl\{ z\in C: F(z,y)+\frac{1}{r}\langle y-z, z-x \rangle\geq0, \forall y\in C \biggr\} , \quad\forall x\in H. $$

*Then the following hold*:

(i) $T_{r}$ *is single*-*valued*;
(ii) $T_{r}$ *is firmly nonexpansive*, *i*.*e*.,
  $$ \Vert T_{r}x-T_{r}y \Vert ^{2}\leq\langle T_{r}x-T_{r}y, x-y\rangle, \quad\forall x,y\in H; $$
(iii) $\operatorname {Fix}(T_{r})=\operatorname {EP}(F)$;
(iv) $\operatorname {EP}(F)$ *is closed and convex*.

Applying Theorem 3.1, Lemma 4.1 and Lemma 4.2, we get the following results.

### Theorem 4.3

*Let* $H_{1}$ *and* $H_{2}$ *be real Hilbert spaces*. *Let* *C* *be a nonempty closed convex subset of* $H_{1}$ *and* *Q* *be a nonempty closed convex subset of* $H_{2}$. *Let* $A:H_{1}\rightarrow H_{2}$ *be a bounded linear operator such that* $A\neq0$, *let* $f:C\rightarrow H_{1}$ *be a monotone and* *k*-*Lipschitz continuous mapping and* $F:Q\times Q\rightarrow\mathbb{R}$ *be a bifunction satisfying* (A1)-(A4). *Setting* $\Gamma=\{z\in \operatorname {VI}(C,f): Az\in \operatorname {EP}(F)\}$, *assume that* $\Gamma\neq\emptyset$. *Let the sequences* $\{x_{n}\}$, $\{y_{n}\}$ *and* $\{t_{n}\}$ *be generated by* $x_{1}=x\in C$ *and*

4.2 $$ \textstyle\begin{cases} y_{n}=P_{C}(x_{n}-\gamma_{n}A^{*}(I-T_{r})Ax_{n}),\\ t_{n}=P_{C}(y_{n}-\lambda_{n}f(y_{n})),\\ x_{n+1}=P_{C}(y_{n}-\lambda_{n}f(t_{n})), \end{cases} $$

*for each* $n\in\mathbb{N}$, *where* $\{\gamma_{n}\}\subset[a,b]$ *for some* $a,b\in(0,\frac{1}{ \Vert A \Vert ^{2}})$ *and* $\{\lambda_{n}\} \subset[c,d]$ *for some* $c,d\in(0,\frac{1}{k})$, $T_{r}$ *is a resolvent of* *F* *for* $r>0$. *Then the sequence* $\{x_{n}\}$ *converges weakly to a point* $z\in\Gamma$, *where* $z=\lim_{n\rightarrow \infty} P_{\Gamma}x_{n}$.

### Proof

Putting $T=T_{r}$ in Theorem 3.1, we get the desired result by Lemmas 4.1 and 4.2. □

The following theorems are related to the zero points of maximal monotone mappings.

### Theorem 4.4

*Let* $H_{1}$ *and* $H_{2}$ *be real Hilbert spaces*. *Let* *C* *be a nonempty closed convex subset of* $H_{1}$ *and* *Q* *be a nonempty closed convex subset of* $H_{2}$. *Let* $A:H_{1}\rightarrow H_{2}$ *be a bounded linear operator such that* $A\neq0$, *let* $f:C\rightarrow H_{1}$ *be a monotone and* *k*-*Lipschitz continuous mapping and* $B:H_{2}\rightarrow2^{H_{2}}$ *be a maximal monotone mapping with* $D(B)\neq\emptyset$. *Setting* $\Gamma=\{z\in \operatorname {VI}(C,f): Az\in B^{-1}0\}$, *assume that* $\Gamma\neq\emptyset$. *Let the sequences* $\{x_{n}\}$, $\{y_{n}\}$ *and* $\{t_{n}\}$ *be generated by* $x_{1}=x\in C$ *and*

4.3 $$ \textstyle\begin{cases} y_{n}=P_{C}(x_{n}-\gamma_{n}A^{*}(I-J_{r})Ax_{n}),\\ t_{n}=P_{C}(y_{n}-\lambda_{n}f(y_{n})),\\ x_{n+1}=P_{C}(y_{n}-\lambda_{n}f(t_{n})), \end{cases} $$

*for each* $n\in\mathbb{N}$, *where* $\{\gamma_{n}\}\subset[a,b]$ *for some* $a,b\in(0,\frac{1}{ \Vert A \Vert ^{2}})$ *and* $\{\lambda_{n}\} \subset[c,d]$ *for some* $c,d\in(0,\frac{1}{k})$, $J_{r}$ *is a resolvent of* *B* *for* $r>0$. *Then the sequence* $\{x_{n}\}$ *converges weakly to a point* $z\in\Gamma$, *where* $z=\lim_{n\rightarrow \infty} P_{\Gamma}x_{n}$.

### Proof

Putting $T=J_{r}$ in Theorem 3.1, we get the desired result. □

### Theorem 4.5

*Let* $H_{1}$ *and* $H_{2}$ *be real Hilbert spaces*. *Let* *C* *be a nonempty closed convex subset of* $H_{1}$ *and* *Q* *be a nonempty closed convex subset of* $H_{2}$. *Let* $A:H_{1}\rightarrow H_{2}$ *be a bounded linear operator such that* $A\neq0$, $f:C\rightarrow H_{1}$ *be a monotone and* *k*-*Lipschitz continuous mapping*, $B:H_{2}\rightarrow2^{H_{2}}$ *be a maximal monotone mapping with* $D(B)\neq\emptyset$ *and* $F:H_{2}\rightarrow H_{2}$ *be an* *α*-*inverse strongly monotone mapping*. *Setting* $\Gamma=\{z\in \operatorname {VI}(C,f): Az\in(B+F)^{-1}0\}$, *assume that* $\Gamma\neq\emptyset$. *Let the sequences* $\{x_{n}\}$, $\{y_{n}\}$ *and* $\{t_{n}\}$ *be generated by* $x_{1}=x\in C$ *and*

4.4 $$ \textstyle\begin{cases} y_{n}=P_{C}(x_{n}-\gamma_{n}A^{*}(I-J_{r}(I-rF))Ax_{n}),\\ t_{n}=P_{C}(y_{n}-\lambda_{n}f(y_{n})),\\ x_{n+1}=P_{C}(y_{n}-\lambda_{n}f(t_{n})), \end{cases} $$

*for each* $n\in\mathbb{N}$, *where* $\{\gamma_{n}\}\subset[a,b]$ *for some* $a,b\in(0,\frac{1}{ \Vert A \Vert ^{2}})$ *and* $\{\lambda_{n}\} \subset[c,d]$ *for some* $c,d\in(0,\frac{1}{k})$, $J_{r}$ *is a resolvent of* *B* *for* $r\in (0,2\alpha)$. *Then the sequence* $\{x_{n}\}$ *converges weakly to a point* $z\in\Gamma$, *where* $z=\lim_{n\rightarrow \infty} P_{\Gamma}x_{n}$.

### Proof

We can easily prove that $z\in(B+F)^{-1}0$ if and only if $z=J_{r}(I-rF)z$ and $J_{r}(I-rF)$ is nonexpansive. Putting $T=J_{r}(I-rF)$ in Theorem 3.1, we get the desired result. □

For a nonempty closed convex subset *C*, the constrained convex minimization problem is to find $x^{*}\in C$ such that

4.5 $$ \phi \bigl(x^{*} \bigr)=\min_{x\in C} \phi(x), $$

where *C* is a nonempty closed convex subset of a real Hilbert space *H* and *ϕ* is a real-valued convex function. The set of solutions of constrained convex minimization problem (4.5) is denoted by $\arg\min_{x\in C}\phi(x)$.

### Lemma 4.6

*Let* *H* *be a real Hilbert space and let* *C* *be a nonempty closed convex subset of* *H*. *Let* *ϕ* *be a convex function of* *H* *into* $\mathbb{R}$. *If* *ϕ* *is differentiable*, *then* *z* *is a solution of* (4.5) *if and only if* $z\in \operatorname {VI}(C,\nabla\phi)$.

### Proof

Let *z* be a solution of (4.5). For each $x\in C$, $z+\lambda(x-z)\in C, \quad\forall\lambda\in(0,1)$. Since *ϕ* is differentiable, we have

$$ \bigl\langle \nabla\phi(z), x-z \bigr\rangle =\lim_{\lambda\rightarrow0^{+}} \frac{\phi(z+\lambda(x-z))-\phi(z)}{\lambda}\geq0. $$

Conversely, if $z\in \operatorname {VI}(C,\nabla\phi)$, i.e., $\langle\nabla\phi(z), x-z\rangle\geq0, \quad\forall x\in C$. Since *ϕ* is convex, we have

$$ \phi(x)\geq\phi(z)+ \bigl\langle \nabla\phi(z),x-z \bigr\rangle \geq\phi(z). $$

Hence *z* is a solution of (4.5). □

Applying Theorem 3.3 and Lemma 4.6, we obtain the following result.

### Theorem 4.7

*Let* $H_{1}$ *and* $H_{2}$ *be real Hilbert spaces*. *Let* *C* *be a nonempty closed convex subset of* $H_{1}$ *and* *Q* *be a nonempty closed convex subset of* $H_{2}$. *Let* $A:H_{1}\rightarrow H_{2}$ *be a bounded linear operator such that* $A\neq0$, *let* $f:C\rightarrow H_{1}$ *be a monotone and* *k*-*Lipschitz continuous mapping and* $\phi:H_{2}\rightarrow\mathbb{R}$ *be a differentiable convex function*, *and suppose that* ∇*ϕ* *is* *α*-*ism*. *Setting* $\Gamma=\{z\in \operatorname {VI}(C,f): Az\in\arg\min_{y\in Q}\phi(y)\}$, *assume that* $\Gamma\neq\emptyset$. *Let the sequences* $\{x_{n}\}$, $\{y_{n}\}$ *and* $\{t_{n}\}$ *be generated by* $x_{1}=x\in C$ *and*

4.6 $$ \textstyle\begin{cases} y_{n}=P_{C}(x_{n}-\gamma_{n}A^{*}(I-P_{Q}(I-\mu\nabla\phi ))Ax_{n}),\\ t_{n}=P_{C}(y_{n}-\lambda_{n}f(y_{n})),\\ x_{n+1}=P_{C}(y_{n}-\lambda_{n}f(t_{n})), \end{cases} $$

*for each* $n\in\mathbb{N}$, *where* $\{\gamma_{n}\}\subset[a,b]$ *for some* $a,b\in(0,\frac{1}{ \Vert A \Vert ^{2}})$ *and* $\{\lambda_{n}\} \subset[c,d]$ *for some* $c,d\in(0,\frac{1}{k})$, $\mu\in(0,2\alpha)$. *Then the sequence* $\{x_{n}\}$ *converges weakly to a point* $z\in\Gamma$, *where* $z=\lim_{n\rightarrow \infty} P_{\Gamma}x_{n}$.

### Proof

Putting $g=\nabla\phi$ in Theorem 3.3, we get the desired result by Lemma 4.6. □

Applying Theorem 3.3 and Lemma 4.6, we obtain the following result. The problem to solve in the following theorem is called split minimization problem (SMP). It is also important in nonlinear analysis and optimization.

### Theorem 4.8

*Let* $H_{1}$ *and* $H_{2}$ *be real Hilbert spaces*. *Let* *C* *be a nonempty closed convex subset of* $H_{1}$ *and* *Q* *be a nonempty closed convex subset of* $H_{2}$. *Let* $A:H_{1}\rightarrow H_{2}$ *be a bounded linear operator such that* $A\neq0$, $\phi_{1}$ *and* $\phi_{2}$ *be differentiable convex functions of* $H_{1}$ *into* $\mathbb{R}$ *and* $H_{2}$ *into* $\mathbb{R}$, *respectively*. *Suppose that* $\nabla\phi_{1}$ *is* *k*-*Lipschitz continuous and* $\nabla \phi_{2}$ *is* *α*-*ism*. *Setting* $\Gamma=\{z\in\arg\min_{x\in C}\phi_{1}(x): Az\in\arg\min_{y\in Q}\phi_{2}(y)\}$, *assume that* $\Gamma\neq\emptyset$. *Let the sequences* $\{x_{n}\}$, $\{y_{n}\}$ *and* $\{t_{n}\}$ *be generated by* $x_{1}=x\in C$ *and*

4.7 $$ \textstyle\begin{cases} y_{n}=P_{C}(x_{n}-\gamma_{n}A^{*}(I-P_{Q}(I-\mu\nabla\phi _{2}))Ax_{n}),\\ t_{n}=P_{C}(y_{n}-\lambda_{n}\nabla\phi_{1}(y_{n})),\\ x_{n+1}=P_{C}(y_{n}-\lambda_{n}\nabla\phi_{1}(t_{n})), \end{cases} $$

*for each* $n\in\mathbb{N}$, *where* $\{\gamma_{n}\}\subset[a,b]$ *for some* $a,b\in(0,\frac{1}{ \Vert A \Vert ^{2}})$ *and* $\{\lambda_{n}\} \subset[c,d]$ *for some* $c,d\in(0,\frac{1}{k})$, $\mu\in(0,2\alpha)$. *Then the sequence* $\{x_{n}\}$ *converges weakly to a point* $z\in\Gamma$, *where* $z=\lim_{n\rightarrow \infty} P_{\Gamma}x_{n}$.

### Proof

Since $\phi_{1}$ is convex, we can easily obtain that $\nabla\phi_{1}$ is monotone. Putting $f=\nabla\phi_{1}$ and $g=\nabla\phi_{2}$ in Theorem 3.3, we obtain the desired result by Lemma 4.6. □

## Conclusion

It should be pointed out that the variational inequality problem and the split feasibility problem are important in nonlinear analysis and optimization. Censor et al. have recently introduced an algorithm which solves the split feasibility problem generated from the variational inequality problem and the split feasibility problem. The base of this paper is the work done by Censor et al. combined with Byrne’s *CQ* algorithm for solving the split feasibility problem and Korpelevich’s extragradient method for solving the variational inequality problem with a monotone mapping. The main aim of this paper is to propose an iterative method to find an element for solving a class of split feasibility problems under weaker conditions. As applications, we obtain some new weak convergence theorems by using our weak convergence result to solve the related problems in a Hilbert space.

Theorem 3.3 improves and extends Theorem 1.1 in the following ways:

(i) The inverse strongly monotone mapping *f* is extended to the case of a monotone and Lipschitz continuous mapping.
(ii) The fixed coefficient *γ* is extended to the case of a sequence $\{\gamma_{n}\}$.
(iii) (1.8) is not necessary in Theorem 3.3.
